# Relationship among cancer treatment, quality of life, and oral function in head and neck cancer survivors: A cross-sectional study

**DOI:** 10.1007/s00520-024-09015-y

**Published:** 2024-11-20

**Authors:** Aya Yokoi, Takayuki Maruyama, Reiko Yamanaka, Noriko Takeuchi, Manabu Morita, Daisuke Ekuni

**Affiliations:** 1 Department of Preventive Dentistry, Faculty of Medicine, Dentistry and Pharmaceutical Sciences, Okayama University, 2-5-1 Shikata-Cho, Kita-Ku, Okayama, 700-8558 Japan; 2 Department of Preventive Dentistry, Division of Dentistry, Okayama University Hospital, Okayama, Japan; 3 Department of Oral Health Sciences, Takarazuka University of Medical and Health Care, Takarazuka, Hyogo Japan

**Keywords:** Quality of life, Oral function, Head and neck cancer, ODK, Tongue pressure

## Abstract

**Purpose:**

Treatment for head and neck cancer (HNC), such as surgery and chemoradiotherapy, can reduce oral function and affect quality of life (QoL). However, whether HNC treatment affects QoL via the decline of oral function remains unclear. This study aimed to investigate the relationship among cancer treatment, QoL, and actual oral function in HNC survivors.

**Methods:**

A total of 100 HNC survivors who had completed definitive treatment for HNC at least 6 months prior to enrollment were enrolled in this cross-sectional study. QoL was evaluated using the European Organization for Research and Treatment of Cancer Quality of Life Questionnaire-Core 30 summary score. Oral diadochokinesis (ODK), tongue pressure, moisture level on the mucosal surface, and mouth opening were measured. Information on age, sex, tumor site, tumor stage, history of HNC treatment, height, body weight, and lifestyle were collected from medical records. Structural equation modeling (SEM) was conducted to analyze the indirect/direct associations among HNC treatment, QoL, and oral function.

**Results:**

In total, 100 HNC survivors (58 males and 42 females; age range, 30–81 years, median, 67 years) were analyzed. Overall, 63 patients (63.0%) were diagnosed as oral cancer, 66 (66.0%) developed advanced cancer (stage 3/4), and 58 (58.0%) underwent reconstruction surgery in 100 HNC survivors. The SEM results supported the hypothesized structural model (root mean square error of approximation = 0.044, comparative fit index = 0.990, Tucker-Lewis index = 0.986). Surgery with neck dissection and reconstruction for advanced cancer had indirect effects on lower QoL via ODK and mouth opening.

**Conclusion:**

HNC treatment is indirectly associated with QoL via oral function in HNC survivors.

## Introduction

Head and neck cancer (HNC) includes malignancies in the oral cavity, mucosal lip, oropharynx, hypopharynx, larynx, and salivary glands, and is the 6th most common cancer worldwide [1]. In 2018, nearly 700,000 new cases of HNC were reported globally [2]. Advances in HNC treatment have increased the number of patients living with HNC [3]. Therefore, needs and concerns are increasing in terms of the provision of long-term support for HNC survivors.

In HNC survivors, surgery and chemoradiotherapy (CRT) have a major impact on oral function and quality of life (QoL). Oral function including speech, swallowing and masticatory performance, and QoL can become impaired after HNC treatment [4–7]. Furthermore, decreased tongue strength, trismus, and hyposalivation have been shown to be correlated with decreased QoL and reductions in both speech and swallowing performance after HNC treatment [5, 8, 9]. However, these associations have been investigated independently, such as the relationships between HNC treatment and oral function and between HNC treatment and QoL. Furthermore, most studies have been based on self-reported as opposed to objective measures of oral function. Therefore, the association between HNC treatment and both QoL and objective oral function remains unknown. Of course, the effects of HNC treatment should be evaluated according to both QoL and posttreatment functional outcomes [10, 11].

Some methodological aspects have improved the quality of research aiming to make a causal inference, such as prospective designs and the use of analytical tools that consider potential confounders. Above all, structural equation modeling (SEM) has been used to estimate changes in QoL and the discrepancy of QoL as an outcome variable to consider the relationships among HNC treatment, QoL, and oral function. Furthermore, the structural regression model in SEM is a combination of measurement model and path model that allows researchers to model explicitly not only complex relations between variables, such as mediation, but also measurement errors [12, 13].

Given this background, we hypothesized that HNC treatment would affect posttreatment QoL through poor oral function. Therefore, the aim of this study was to investigate the association among HNC treatment, QoL, and actual oral function in HNC survivors using SEM.

## Material and methods

### Ethics statement

The study protocol of this cross-sectional study was approved by the ethics committee of Okayama University Hospital (No. 1810–034, October 12, 2018). All procedures were conducted in accordance with the ethical standards of the responsible committees on human experimentation (institutional and national) and with the Helsinki Declaration of 1964 and later versions. Written informed consent was obtained from all patients for inclusion in the study. This study conformed with the Strengthening the Reporting of Observational Studies in Epidemiology (STROBE) guidelines for cross-sectional studies [14].

### Study population

The cross-sectional study recruited and assessed outpatients who were receiving oral hygiene care from the Clinical Division of Preventive Dentistry at Okayama University Hospital, Japan, from November 2018 to December 2021. These patients periodically received the professional oral hygiene care by dentist and dental hyginists, including oral hygiene instructions, professional toothbrushing, scaling and root planning from the perioperative period to the recruitment. The inclusion criteria were as follows: 1) outpatients with a history of HNC who were receiving oral hygiene care and 2) the ability to provide written informed consent. Patients had completed definitive treatment for HNC at least 6 months prior to enrollment when both the QoL score evaluated by self-questionnaire and oral function measured by speech therapists were stabilized [15, 16]. The exclusion criteria were: 1) inability to continue the study because of severe malaise; 2) loss of speaking and swallowing function due to laryngectomy and/or neuromuscular disease; 3) declining to agree to participate in the study; and 4) incomplete data. The data of QoL, oral function, and HNC treatment were collected at the same time when they visited the Clinical Division of Preventive Dentistry.

### Evaluation of QoL

Patients were asked to report their QoL using the European Organization for Research and Treatment of Cancer Quality of Life Questionnaire-Core 30 (EORTC QLQ-C30) when they visited the Clinical Division of Preventive Dentistry for receiving oral hygiene care. EORTC QLQ-C30 has been widely used in cancer studies [17, 18]. We used the EORTC QLQ-C30 summary score for evaluating QoL in HNC survivors, which was used in the previous study [16]. The EORTC QLQ-C30 is a 30-item self-report measure on health status, functioning, and symptoms among individuals with cancer in clinical trials. The 30 items are categorized into 15 subscales. A subscale score is the mean value of some items, and is showed value from 0 to 100. The summary score is calculated from the mean value of 13 subscales (among the 15 subscales, global health status and the financial difficulties scale were excluded) according to the EORTC QLQ-C30 scoring manual [17, 19]. The summary score is showed value from 0 to 100 as overall QoL, which is useful in SEM analysis as continuous variable. Global health status was evaluated using the following items: “How would you rate your overall health during the past week?” and “How would you rate your overall quality of life during the past week?”. The response options were from very poor (1) to excellent (7). Summary score, global health status and functional scales with higher scores indicate good QoL. Symptom scales with low scores indicate good QoL [17].

Missing data were analyzed according to the EORTC QLQ-C30 scoring manual [17]. The following method was used to impute items from multi-item scales: if at least half the items of the one subscale were completed, the missing item was estimated as the mean value of the other items in the same subscale.

### Evaluation of oral function

Tongue pressure, moisture level on the mucosal surface, mouth opening, and oral diadochokinesis (ODK) were measured for the evaluation of oral function. All evaluations were conducted by one trained dentist.

The ODK test evaluates the speed and regularity of articulatory organs by making alternating syllables move as quickly as possible. It is widely used to evaluate motor dysarthria. In Japan, three types of syllables, i.e., “pa”, “ta”, and “ka”, are often used. Of these, “pa” evaluates the function of the lips, “ta” evaluates the function of the tongue tip, and “ka” evaluates the function of the tongue dorsum. The participants were asked to repeat each given syllable, “pa/ta/ka”, sequentially as fast as possible for 5 s using an oral function measuring device (KENKOU-KUN, Takei Scientific Instruments Co., Ltd., Niigata, Japan). The number of repetitions per second was calculated as the repetition speed of the syllables [20].

Tongue pressure measurements were performed using the JMS tongue pressure measurement system (JMS Co. Ltd., Hiroshima, Japan) [21]. The patient was placed in a relaxed sitting position and asked to place the balloon on the anterior part of the palate. The patient was then asked to raise the tongue and compress the balloon onto the palate as much as possible for 7 s.

Moisture level on the mucosal surface was measured at the center of the right buccal mucosa. An oral moisture meter (Mucus^®^, LIFE, Tokyo, Japan) was used for the evaluation of hyposalivation. The meter sensor was pressed on the mucosa with a pressure of approximately 200 g so that it contacted the surface to be measured uniformly, and the measured value was displayed within approximately 2 s [22]. All measurements were repeated three times, and the mean value was used as the individual moisture level.

Mouth opening was recorded as the distance between the maxillary and mandibular incisors using a millimeter ruler. When the patient was edentulous or partially edentulous, the measurement was performed with the dentures. If the patient did not wear dentures, the measurement was made between the maxillary and mandibular alveolar ridges, followed by the subtraction of the mean crown length of the maxillary (9 mm) and mandibular (8 mm) incisors [5].

### General status assessment

Medical charts were reviewed to obtain information about the participants’ age, sex, body weight, body mass index, and several other type of HNC information, including tumor site, tumor stage (International Union Against Cancer ver. 7), time since completed treatment, type of treatments, with or without reconstruction, and with or without neck dissection. The information about lifestyle was extracted from the patients’ medical charts. Lifestyle information included employment status, smoking habits, and drinking habits. Smoking status was categorized into “never”, “past”, and “current” [23]. These data of general status were collected at the same time with QoL and oral function, when they visited the Clinical Division of Preventive Dentistry. The information about body weight and lifestyle was at the time of evaluating QoL and oral function.

### Statistical analysis

**Fig. 1:**
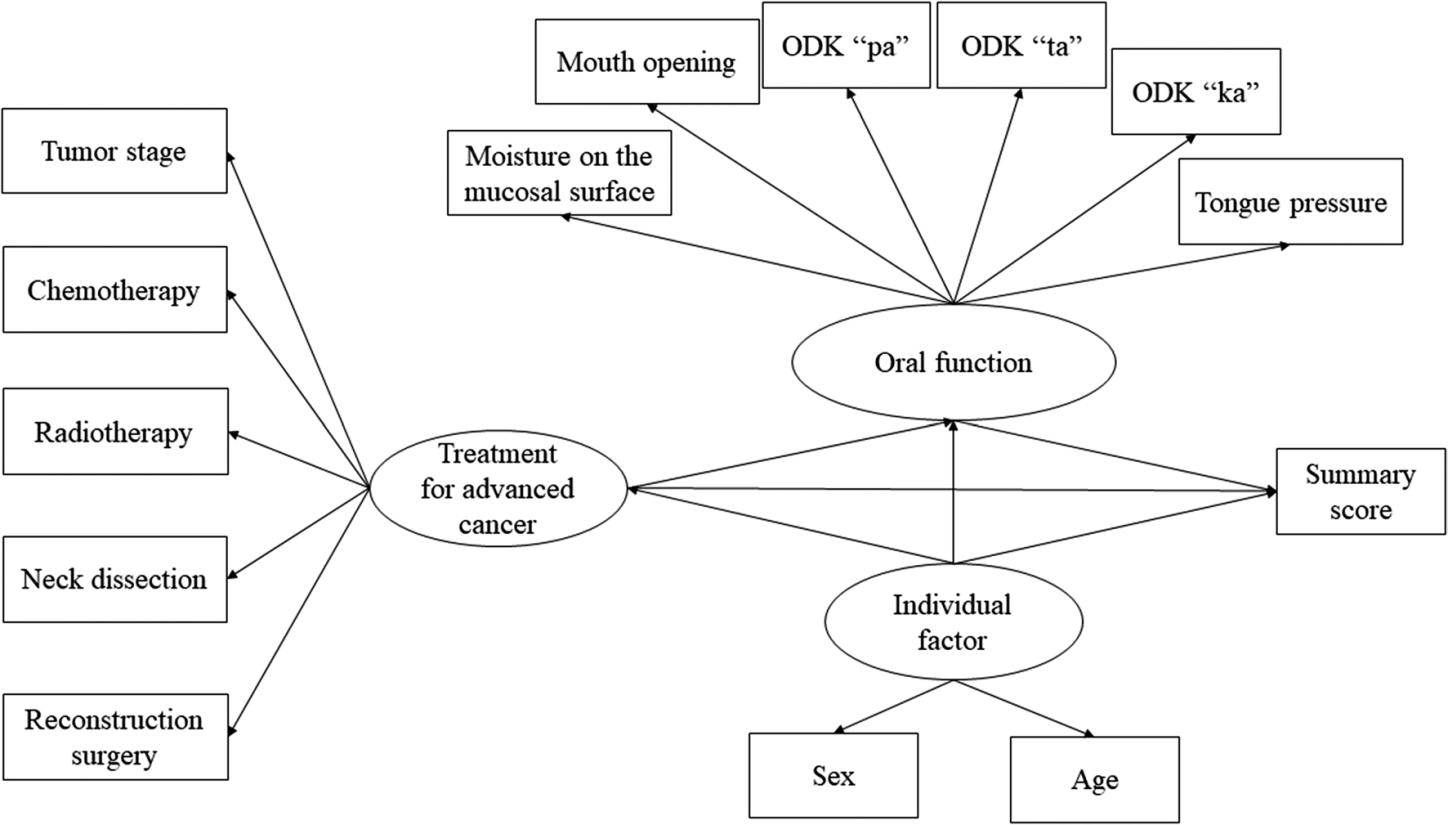
Hypothesis model of factors influencing QoL in HNC survivors (QoL: quality of life; HNC: head and neck cancer)

The normality of data was investigated by the Shapiro-Wilk test [24]. Structural equation modeling (SEM) is a powerful analytic method. Several studies have often utilized SEM to examine if hypothesized conceptual models and structural relationships at the conceptualization stage are supported by the empirical data provided by their study sample [12, 13]. The sample size was estimated from a previous study [25] suggesting that a sample size of at least 100 was needed in SEM analysis. This study suggested also that the part of solutions was problematic when sample size was very small (20 or 50) and reliability was low (α = 0.60), and that non-convergence and improper values are frequent occurrences at sample sizes under 100 [25]. Logical associations were analyzed using SEM analysis. SEM was performed to create a path diagram, and relationships among cancer treatment, QoL, and actual oral function in HNC survivors were clarified rather than simply checking the correlation [26]. Figure 1 shows an ideal model based on our hypothesis, which was estimated based on previous studies [5, 8, 11, 20, 27–33]. This model was developed on the basis of the model of Wilson and Cleary [33]. We assumed that HNC treatment was related directly and/or indirectly to QoL in HNC survivors. The present study included continuous, dichotomous, and categorical data. Therefore, weighted least-squares parameter estimates were selected. Data analysis was performed with SEM using statistical software (Mplus version 8.0; Muthen & Muthen, Los Angeles, CA, USA). The goodness of fit of the model was assessed using the root mean square error of approximation (RMSEA), the comparative fit index (CFI), and the Tucker-Lewis index (TLI). An RMSEA value < 0.08 suggested an adequate fit, whereas CFI and TLI represented an incremental fit; values > 0.95 indicated an adequate fit, whereas those > 0.90 were still acceptable. Non-significant paths were removed step-by-step after confirmatory factor analysis [34]. Values of p < 0.05 were considered significantly paths [35].

SPSS (version 25; IBM, Tokyo, Japan) was used for the statistical analysis except for SEM. The association between QoL and general status was analyzed using the Mann-Whitney *U* test. The patients were divided into groups as follows: age ≥ 65 and < 65, male and female, oral cancer (tongue, maxilla and mandible) and non-oral cancer (sinuses, salivary glands, nasopharynx, oropharynx, hypopharynx and larynx), advanced cancer (stage = 3, 4) and early cancer (stage = 1, 2, other), ≥ 5 and < 5 years from treatment, radiotherapy ( +) and (–) groups, chemotherapy ( +) and (–) groups, reconstruction ( +) and (–) groups, and neck dissection ( +) and (–) groups. QoL was also compared between the oral function low and high groups using the Mann-Whitney *U* test. The patients were dichotomized into two groups on the basis of oral function: tongue pressure ≥ 30 and < 30 [8], moisture level on the mucosal surface ≥ 28 and < 28 [36], mouth opening ≥ 35 and < 35 [5], and ODK “pa” ≥ 6 and < 6, ODK “ta” ≥ 6 and < 6, ODK “ka” ≥ 6 and < 6 [20]. The level of significance was set at p < 0.05.

## Results

**Fig. 2:**
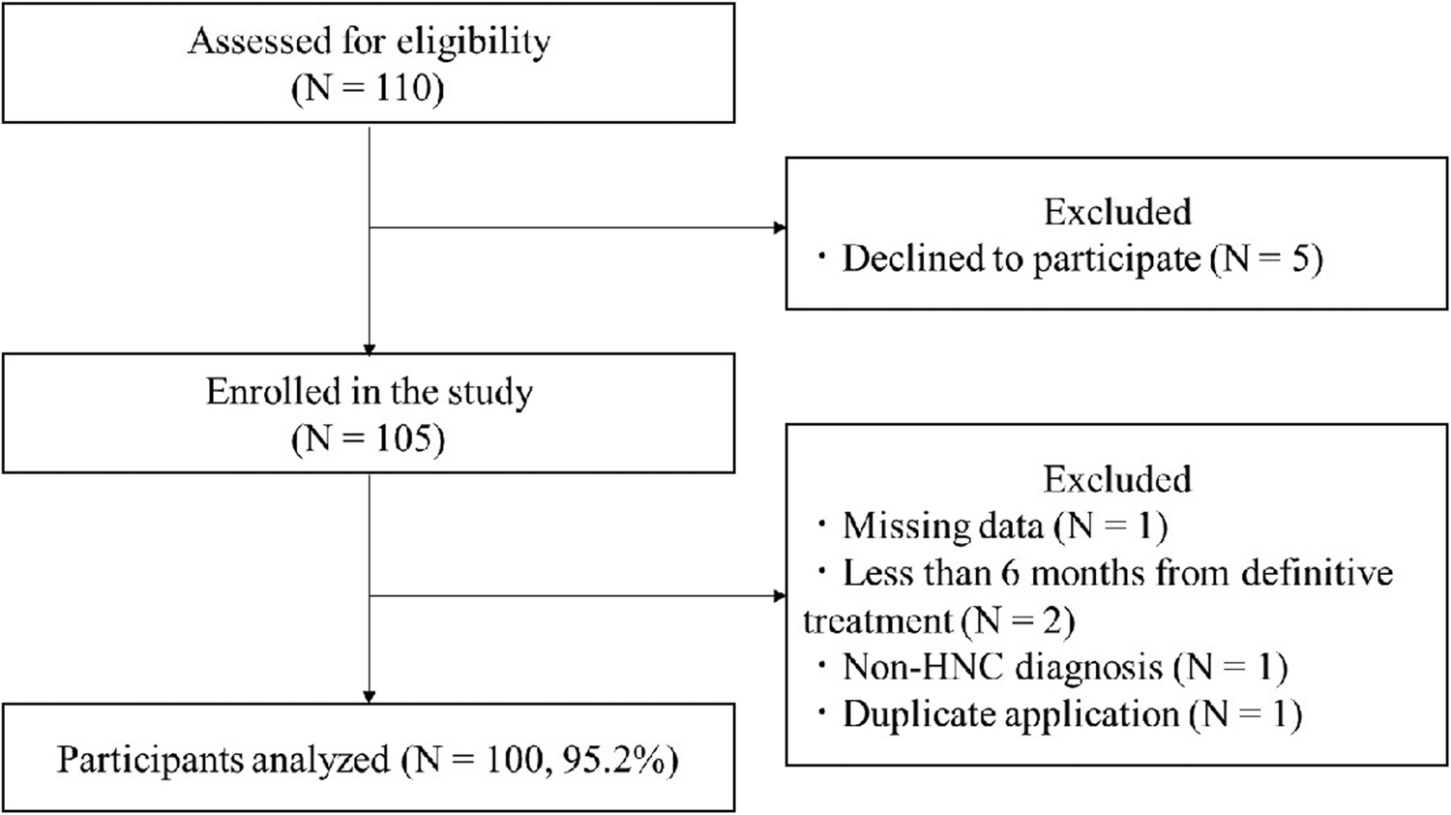
Recruitment flowchart

As shown in Fig. 2, of the 110 patients, 100 (58 males and 42 females; age range, 30–81 years, median age, 67 [median] and 64 [mean] years) completed this study. Five patients were excluded from the analysis in this study because they had severe malaise and missing data. The characteristics of the patients are shown in Table 1. All patients have finished HNC treatment, and recovered completely from HNC at least 6 months prior to enrollment. Overall, 63 patients (63.0%) were diagnosed as oral cancer, 66 (66.0%) developed advanced cancer (stage 3 or 4), and 58 (58.0%) underwent reconstruction surgery as a past medical history.

**Table 1:** Characteristics of the participants (N = 100)

Variable		Median (25%, 75%) / N (%)
Age (years)		67 (56, 71)
Sex	Male	58 (58.0)
	Female	42 (42.0)
Weight (kg)		55.3 (47.9, 63.0)
Body mass index (kg/m^2^)		21.0 (19.1, 23.4)
Tumor site	Sinuses	1 (1.0)
	Oral cavity	63 (63.0)
	Salivary glands	6 (6.0)
	Nasopharynx	3 (3.0)
	Oropharynx	9 (9.0)
	Hypopharynx	8 (8.0)
	Larynx	6 (6.0)
	Other	4 (4.0)
Tumor stage	1	10 (10.0)
	2	20 (20.0)
	3	15 (15.0)
	4	51 (51.0)
	Other	4 (4.0)
Time since completed treatment	< 1 year	13 (13.0)
	1–5 years	47 (47.0)
	5–10 years	31 (31.0)
	≥ 10 years	9 (9.0)
Type of treatment	Surgery only	18 (18.0)
	Radiotherapy only	1 (1.0)
	Surgery and chemotherapy	20 (20.0)
	Surgery and radiotherapy	6 (6.0)
	Chemotherapy and radiotherapy	19 (19.0)
	All (surgery, chemoradiotherapy)	36 (36.0)
Reconstruction	( +)	58 (58.0)
Neck dissection	( +)	65 (65.0)
Employed currently	( +)	39 (39.0)
Tube feeding	( +)	5 (5.0)
Smoking status	Never	47 (47.0)
	Past	53 (53.0)
	Current	0 (0.0)
Drinking status	Drinker	67 (67.0)
	Nondrinker	33 (33.0)

The oral function and QoL of the patients are shown in Table 2. Tongue pressure (median [25%, 75%] and mean ± standard deviation [SD]) was 24.6 (15.9, 32.2) and 24.7 ± 11.4 kPa. The EORTC QLQ-C30 summary score (median [25%, 75%] and mean ± SD) was 90.5 (81.2, 96.2) and 88.1 ± 10.2.

**Table 2:** Oral function and QoL scores of the patients (N = 100)

Variable	Median (25%, 75%)
Tongue pressure (kPa)	24.6 (15.9, 32.2)
Moisture on the mucosal surface	29.1 (27.9, 30.1)
Mouth opening (mm)	40.0 (34.5, 45.0)
Oral diadochokinesis (times/s)	
“Pa”	5.6 (5.0, 6.2)
“Ta”	5.4 (4.6, 6.1)
“Ka”	5.2 (4.4, 5.8)
EORTC QLQ-C30 summary score	
Summary score	90.5 (81.2, 96.2)
Global health status	66.7 (50.0, 83.3)
Functional scales	
Physical functioning	93.3 (86.7, 100)
Role functioning	100 (83.3, 100)
Emotional functioning	91.7 (83.3, 100)
Cognitive functioning	83.3 (66.7, 100)
Social functioning	100 (83.3, 100)
Symptom scales	
Fatigue	22.2 (11.1, 33.3)
Nausea and vomiting	0 (0, 0)
Pain	0 (0, 20.8)
Dyspnoea	0 (0, 33.3)
Insomnia	0 (0, 33.3)
Appetite loss	0 (0, 0)
Constipation	0 (0, 33.3)
Diarrhoea	0 (0, 33.3)
Financial difficulties	0 (0, 33.3)

*QoL*: quality of life; *EORTC QLQ-C30*: European Organization for Research and Treatment of Cancer Quality of Life Questionnaire-Core 30

**Fig. 3:**
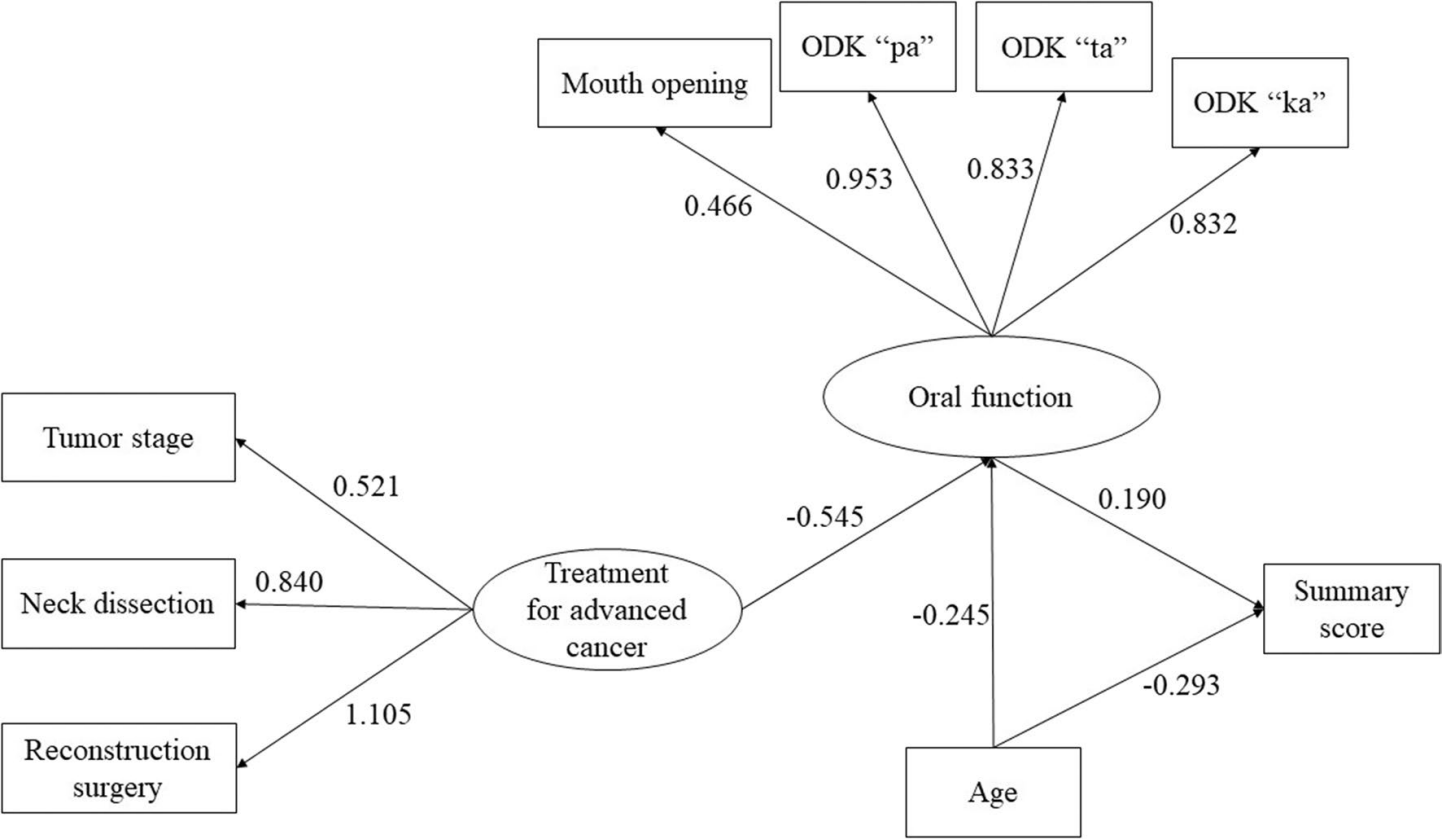
Finalized model of factors affecting QoL in HNC survivors. Ovals indicate latent variables, squares indicate observed variables, and numbers on the arrows are standardized coefficients of influence, with signs indicating enhancing or reducing effects. The SEM results supported the final structural model (RMSEA = 0.044, CFI = 0.990, TLI = 0.986) (QoL: quality of life; HNC: head and neck cancer; RMSEA: root mean square error of approximation; CFI: comparative fit index; TLI: Tucker-Lewis index)

As a result, the hypothesis model was modified on the basis of confirmatory factor analysis. ODK and mouth opening for oral function factor fit the specified factor structure (RMSEA = 0.071, CFI = 0.996, TLI = 0.988). The SEM results supported the final structural model after confirmatory factor analysis (RMSEA = 0.044, CFI = 0.990, TLI = 0.986) (Fig. 3). In Fig. 3, ovals indicate latent variables, squares indicate observed variables, the numbers on the arrows are standardized coefficients of influence, standardized coefficients > 0 indicate a positive correlation, and standardized coefficients < 0 indicate a negative correlation. All pathways were significant (p < 0.05). Surgery with neck dissection and reconstruction for advanced cancer had also indirect effects on QoL via poor oral function.

Table 3 shows the effects of HNC treatment and oral function on EORTC QLQ-C30 summary scores. Advanced age and having undergone radiotherapy were significantly related to lower summary scores (p = 0.005 and p = 0.004, respectively). The EORTC QLQ-C30 summary score was lower in the low-score ODK “pa” group than in the high-score group (p = 0.022). No significant differences in summary scores were observed with respect to other types of HNC treatment and oral function (Table 3).

**Table 3:** Relationships among summary scores, general status, and oral function

Variable		Summary score	*p* value^a^
Age, years	≥ 65, n = 59	89.7 (78.1, 95.0)^b^	0.005
	< 65, n = 41	94.0 (86.1, 98.4)	
Sex	Female, n = 42	89.8 (78.3, 95.5)	0.259
	Male, n = 58	91.7 (83.0, 96.7)	
Tumor site	Oral cavity, n = 63	91.7 (80.8, 96.4)	0.434
	Other, n = 37	88.7 (80.1, 95.0)	
Tumor stage	1, 2, other, n = 34	92.2 (81.3, 96.8)	0.469
	3, 4, n = 66	90.3 (80.0, 96.2)	
Time since completed treatment	≥ 5, n = 40	93.5 (80.7, 96.8)	0.383
	< 5, n = 60	89.9 (80.9, 95.9)	
Radiotherapy	( +) n = 62	88.4 (79.2, 94.0)	0.004
	(–) n = 38	95.1 (87.6, 98.2)	
Chemotherapy	( +) n = 75	98.3 (79.4, 95.3)	0.063
	(–) n = 25	95.0 (85.5, 97.8)	
Reconstruction	( +) n = 58	90.5 (81.4, 96.5)	0.842
	(–) n = 42	90.7 (78.5, 96.0)	
Neck dissection	( +) n = 65	90.5 (81.0, 96.3)	0.968
	(–) n = 35	90.3 (79.3, 96.2)	
Tongue pressure (kPa)	≥ 30, n = 35	93.8 (82.6, 98.5)	0.114
	< 30, n = 65	89.7 (79.1, 95.6)	
Moisture on the mucosal surface	≥ 28, n = 71	90.2 (80.2, 96.2)	0.630
	< 28, n = 29	93.4 (81.5, 95.9)	
Mouth opening (mm)	≥ 35, n = 74	92.0 (84.5, 96.5)	0.064
	< 35, n = 26	83.7 (76.7, 96.2)	
Oral diadochokinesis “pa”	≥ 6, n = 35	95.2 (86.0, 98.2)	0.022
	< 6, n = 65	90.0 (79.0, 95.0)	
Oral diadochokinesis “ta”	≥ 6, n = 30	93.7 (85.6, 98.3)	0.054
	< 6, n = 70	90.1 (78.6, 95.5)	
Oral diadochokinesis “ka”	≥ 6, n = 20	92.8 (85.5, 99.1)	0.114
	< 6, n = 80	90.3 (80.3, 95.8)	

^a^Mann-Whitney *U* test

^b^Median (25%, 75%)

## Discussion

In the present study, we focused on the influence of HNC treatment on oral function and QoL in HNC survivors. To the best of our knowledge, this is the first study to investigate the comprehensive relationship among HNC treatment, actual oral function, and QoL in HNC survivors by using SEM analysis.

In the SEM analysis, reconstruction surgery and neck dissection for advanced cancer were significantly related to poor oral function. This result is similar to that reported in previous studies [5, 9, 31]. Treatment for advanced cancer related to the mastication muscles can cause trismus after HNC treatment [32]. In previous studies, oral function has often been evaluated using self-report questionnaires. By contrast, the present study reports a relationship between HNC treatment and oral function evaluated objectively by a dentist.

In the SEM analysis, trismus and poor ODK were associated with poor QoL. Trismus is associated with compromised speech and poor QoL [5, 32, 37]. ODK was measured as tongue motor function related to dysarthria [22, 38]. Therefore, trismus and poor ODK appear to lead to speaking difficulties. HNC survivors reported speaking as one of the issues with the most impairment [39]. Trismus and poor ODK might affect QoL via difficulty in speaking.

In the SEM analysis, poor oral function was related to a poor QoL as evaluated by the EORTC QLQ-C30 summary score. One study showed that in HNC survivors, jaw exercise therapy increased the maximal interincisal opening and improved QoL [40]. The other oral exercise, which included turning the head, pouting lips, bulging cheeks, stretching tongue, articulation exercise and salivary gland massages, exhibited greater improvement in ODK [41]. This study reported that ODK was showed improvement. Oral exercise may improve not only ODK but also QoL. However, no evidence was seen of effective training for improving ODK and QoL. Further studies about training for ODK are therefore needed to improve QoL in HNC survivors.

A review by von Nieuwenhuizen et al. showed strong evidence for the association between the change in global QoL from pre-treatment to 6 months posttreatment and the survival rate in HNC patients. They suggested that improving QoL may be an interesting intervention to improve survival rates [42]. Rehabilitation for oral function improved QoL [40]. Rehabilitation for oral function by dental staff might be necessary in posttreatment for improving QoL and survival rates, in addition to perioperative oral management. Dental staff should monitor not only oral condition but also oral function and QOL among HNC survivors. Our findings suggest the need to provide dental interventions for long time in HNC survivors after cancer treatment.

Advanced age (≥ 65 years old) was significantly associated with poor QoL in the SEM analysis. This result is opposite to those reported in previous studies [9, 43]. Laraway suggested that older individuals may be better adapted to a poor body image after treatment and are generally likely to be less conscious of body image [44]. In these studies, QoL was evaluated based on mean University of Washington Quality of Life (UW-QoL) instrument subscale scores (functioning, economic status, and symptoms) or the mean cancer-specific QoL score (appearance, economic status, and distress) of Quality of Life in Adult Cancer Survivors (QLAS) [9, 43]. In the present study, QoL was evaluated based on the EORTC QLQ-C30 summary score, which was calculated from the mean subscale scores (health status, functioning, and symptoms). Older people generally have worse oral function. Compared with UW-QoL and QLAS, the EORTC QLQ-C30 summary score may be easily influenced by actual oral function.

Advanced age was significantly associated with a lower QoL score and poor oral function in the SEM analysis. Japan is a super-aging society, with about 30% of its citizens already aged > 65 years [45]. As a result, an increase in older HNC patients and survivors is expected in Japan. Older people were significantly more likely to have poor oral function [46]. Older HNC patients and survivors might have lower oral function and poorer QoL. Therefore, the need for rehabilitation to improve oral function is increasing after HNC treatment to improve QoL.

This study is strengthened by some facts. First, oral function was evaluated by dentist. Most studies have been based on self-reported as opposed to objective measures of oral function. Therefore, the association between HNC treatment and both QoL and objective oral function remained unknown. Second, the patients in the present study may not represent a specific population. Because, the mean tongue pressure (24.7 kPa) was similar to that reported in previous studies (25.5 kPa) of Japanese HNC survivors using the same device [11]. The mean global health status (69.3) (data not shown) was within the range of previous studies (61.6–73.6) using the same questionnaire [11, 47].

However, this study has some limitations. First, this was a cross-sectional study. To clarify the causal relationship, a prospective cohort study and an interventional study would be needed. Second, we did not consider other important confounding factors such as education level, marital status, income and masticatory performance and number of present teeth [5–7, 48].

## Conclusion

The findings of this study suggest that HNC treatment is indirectly associated with QoL via oral function in HNC survivors.

## Data Availability

No datasets were generated or analysed during the current study.
